# Alcohol Consumption and Subclinical Findings on Cognitive Function, Biochemical Indexes, and Cortical Anatomy in Cognitively Normal Aging Han Chinese Population

**DOI:** 10.3389/fnagi.2018.00182

**Published:** 2018-06-19

**Authors:** Lin Sun, Hua Xu, Jie Zhang, Wei Li, Jing Nie, Qi Qiu, Yuanyuan Liu, Yuan Fang, Zhi Yang, Xia Li, Shifu Xiao

**Affiliations:** ^1^Alzheimer’s Disease and Related Disorders Center, Department of Geriatric Psychiatry, Shanghai Mental Health Center, Shanghai Jiao Tong University School of Medicine, Shanghai, China; ^2^Shanghai East Hospital, Key Laboratory of Arrhythmias, Ministry of Education, Tong Ji University School of Medicine, Shanghai, China

**Keywords:** alcohol consumption, light-to-moderated alcohol, cognitive function, biochemical markers, left superiortemporal gyrus

## Abstract

**Background:** Binge drinking of alcohol is associated with brain damage, but less is known about relationship of light-to-moderate alcohol consumption with cognitive function, biochemical indexes, and cortical anatomy. Previous findings have debated on whether light-to-moderate drinking has any health benefits. We investigated cortical thickness and its association with alcohol consumption and cognitive functions in a non-dementia aging Han Chinese population.

**Methods:** 940 non-dementia aging subjects were included in our study (alcohol *n* = 149; non-alcohol *n* = 791). Among them, 572 received blood biochemical tests including liver function and lipid metabolism (alcohol *n* = 100; non-alcohol *n* = 472) and 141 had brain magnetic resonance imaging (alcohol *n* = 27; non-alcohol *n* = 114). The Beijing version of the Montreal Cognitive Assessment and the Chinese version of the neuropsychological test battery were used to assess cognitive functions.

**Results:** There was no significant difference in cognitive functions between alcohol and non-alcohol groups in the overall database. Similarly, there was no significant difference in liver function and lipid metabolism between two groups in the sub-database. The left superiortemporal gyrus was one of age sensitive regions and alcohol consumption was significantly associated with thinner cortex of the left superiotemporal cluster in the sub-database.

**Conclusion:** Alcohol consumption was not significantly associated with better or worse cognitive function and biochemical indexes abnormality, however, significantly associated with thinner cortex of the left superiortemporal gyrus in cognitively normal aging Han Chinese population.

## Introduction

Alcohol is the most widely used addictive substance worldwide, and it has been linked to over 200 diseases and is responsible for 5.9% of global deaths (Clarke et al., 2017). It is well-known that binge drinking of alcohol impairs cognitive control of behavior such as inhibition, top–down response selection, and conflict monitoring (Crews and Boettiger, 2009). Chronic alcohol intoxication results in significant activation of neurodegenerative processes (Vetreno et al., 2017), and alcoholic brains show shrinkage of cortical and subcortical structure (Vetreno and Crews, 2015). However, it is still debatable whether light-to-moderate drinking has any health benefits (Davis et al., 2014; Shokri-Kojori et al., 2017). Corley et al. (2011) included 922 healthy adults about 70 years old with IQ data, and found that alcohol consumption made a small independent contribution to memory performance and verbal ability, although the clinical significance of these findings was uncertain. In contrast, Degerud et al. (2017) demonstrated that light-to-moderate drinking was associated with worse heath characteristics, and a study from the Framingham Offspring Study who underwent brain magnetic resonance (MR) imaging and drinking habit inquiries showed a significant negative linear relationship between alcohol consumption and total cerebral brain volume, which suggested moderate drinking was not protective (Paul et al., 2008). There are many studies about the relationship of drinking alcohol with adolescent brain development, but few with elderly cognition and brain anatomy. Here, because binge drinking has been determined to be harmful, we explored the relationship of light-to-moderate drinking with cognitive function, biochemical indexes, and brain anatomy in a cohort of non-dementia aging Han Chinese population.

## Materials and Methods

### Subjects

This was a cross-sectional investigation conducted in Shanghai supported by the National Pillar Program of China Ministry of Science and Technology (project number: 2009BAI77B03) from 2011 to 2012 (Xiao et al., 2013); 940 cognitively normal aging subjects were included (alcohol *n* = 149; non-alcohol *n* = 791). They underwent a screening process that included medical history, physical and neurological examinations, and cognitive assessments by a face-to-face interview. We recruited 572 of the subjects to receive blood tests (alcohol *n* = 100; non-alcohol *n* = 472), and 141 for brain MR imaging (alcohol *n* = 27; non-alcohol *n* = 114). All subjects also needed to meet the following criteria: (1) Han Chinese, ≥60 years old; (2) absence of dementia; (3) accord with Petersen Mini-Mental State Examination (MMSE) (Folstein et al., 1975) cutoff score, uneducated subjects ≥18, elementary school educated subjects ≥21, and higher than middle-school educated subjects ≥25; (4) without major medical abnormalities, including nervous system disease or unstable, acute or life-threatening medical illness; (5) able to complete the study; and (6) denial of binge alcohol drinking (men < 5 standard units/day and women < 4 standard units/day) (Vinader-Caerols et al., 2017). Individuals with a history of mental disease or other disorders that might affect cognitive function were excluded. The Beijing version of the Montreal Cognitive Assessment (MoCA) was used to assess cognitive function (Nasreddine et al., 2005). This screening test consists of 30 items measuring multiple cognitive domains (including visual space, naming, attention, calculation, abstract, delay recall, orientation, and language function). The Chinese version of the neuropsychological test battery (NTB) was also used in the study (Liu et al., 2011), which detected digit span, auditory verbal learning, associative learning, visual discrimination, language fluency, mapping, and a test with blocks.

We divided the subjects into groups based on their history of alcohol consumption. We defined one standard drink unit as 14 g of pure ethanol (Pilatti et al., 2017). Non-alcohol users were defined as individuals who never drink or rarely drink (such as holiday drinking, lower one standard unit per time). Alcohol users were required to provide types of alcohol drunk (liquor, 40% alcohol; beer, 3% alcohol; wine, 11% alcohol; and yellow rice, 12% alcohol), and daily intake of alcohol, regardless of whether they consumed alcohol at present. Before this study was initiated, all subjects had signed informed consent, and ethical approval had been obtained from the Ethics Committee of the Shanghai Mental Health Center.

### Measurement of Biochemical Indexes

Peripheral blood samples were collected at 7–9 am after an overnight fast. Clot activating gel-containing serum separator tubes were used for testing of biochemical parameters. Plasma triglycerides (TG), cholesterol, high density lipoprotein (HDL), low density lipoprotein (LDL), total bilirubin, total protein, alanine transaminase (ALT), and aspartate transaminase (AST) were measured using an Olympus AU2700 automatic biochemical analyzer (Beckman Coulter, Inc., Carlsbad, CA, United States).

### MR Image Acquisition and Processing

Magnetic resonance images were acquired with a Siemens Magnetom Verio 3.0T scanner (Siemens, Munich, Germany). T_1_-weighted images were obtained with 176 sagittal slices using the 3D magnetization prepared rapid gradient echo acquisition sequence with the following parameters: TR = 2300 ms, TE = 2.98 ms, Flip angle = 9°, spatial resolution = 1 mm × 1 mm × 1.2 mm.

T_1_-weighted images for subjects were processed into surface-based structural data using the automated reconstruction function in the FreeSurfer version 6.0 software described by Dale et al. (1999), which was download online^1^. FreeSurfer was applied to segment brain gray matter, white matter and cerebrospinal fluid and reconstruct brain white-gray matter boundary surface. Individual participants’ cortical thickness, subcortical structure volume, and total intracranial volume were then extracted directly from FreeSurfer. Meanwhile, we conducted a whole brain vertex-wise analysis in Qdec to compare the difference of two groups on cortical thickness measures. A smoothing Gaussian kernel of 10 mm was applied before comparison at the level of each vertex. Multiple comparisons were controlled with a Monte Carlo Null-Z Simulation of 1%.

### Data Analysis

Demographics, lifestyle, physical disease, and cognitive scores were analyzed using variance analysis for continuous variables and a χ^2^ test for the categorical variables between alcohol and no-alcohol groups. The confounding factors were regressed, including demographics, physical disease, and life style indexes. A general linear model was used to analyze the differences in cognitive function scores and biochemical indexes between groups after adjusting for confounding factors.

For balancing the sample size and gender bias in alcohol and non-alcohol groups, a random resampling method was employed 200 times to build up the data sets in the analysis of cognitive function and blood biochemical indexes, respectively. In the analysis of cognitive function, each 20 male samples and 20 female samples were selected from alcohol group and non-alcohol group. In the analysis of blood biochemical indexes, each 12 male samples and 12 female samples were selected from alcohol group and non-alcohol group. Then, variance analysis for continuous variables and a χ^2^ test for the categorical variables between alcohol and no-alcohol groups were performed in each resampling constructed sets. Finally, an average *F* or χ^2^ value and an average *p*-value were calculated after the resampling process.

Correlations of cortical thickness with age in 114 non-alcohol subjects including males and females with MR image data were respectively examined by Pearson’s correlation and linear regression analysis. A general linear model was used to analyze the differences in subcortical structure volume proportion between groups after adjusting for confounding factors. For each vertex, Qdec software (Wallon et al., 2012) was used to detect vertex-based clusters in cortical thickness between groups without females (alcohol *n* = 25, non-alcohol *n* = 49), and Monte Carlo Null-Z Simulation < 0.01 correction was adopted. All statistical analyses used SPSS Version 17.0 software with two-tailed *p-*values of 0.05.

## Results

### Cognitive Functions Between Alcohol and Non-alcohol Consumption Groups in the Overall Database

The prevalence of light-to-moderate alcohol consumption in non-dementia elderly in the Shanghai community examined was 15.85% (149/940). In the alcohol group, 36.2% drank liquor, 15.3% beer, 10.7% wine, and 37.6% yellow rice. The demographics, physical disease, lifestyle, and cognitive scores for the alcohol (*n* = 149) and non-alcohol (*n* = 791) groups are listed in **Table 1**. There were observed differences between groups in demographics, lifestyle, and physical disease, so the effects of those were regressed. Through regular statistical analysis, lower scores for abstract and mapping were found in alcohol drinking group than non-alcohol group (*p* < 0.05), and there were no statistical differences in total MoCA scores or other domains of NTB between two groups (*p* > 0.05). For balancing the gender and sample size bias in two groups, bootstrapping analysis was used and showed that there were no any positive findings (*p* > 0.05).

**Table 1:** Demography, life style, and cognitive scores of the subjects in non-dementia aging Han Chinese population.

	Alcohol (*n* = 149)	Non-alcohol (*n* = 791)	*F* or χ^2^	*p*-Value	Bootstrapping	
					Mean *F* or χ^2^	Mean *p*-value
Age (years)	70.00 ± 6.888	70.25 ± 6.863	0.168	0.682	0.748	0.394
Male/female	129/20	274/517	138.093	0.000*	6.073	0.014*
Education (years)	10.29 ± 3.730	10.73 ± 3.879	1.620	0.203	0.983	0.329
Smoking (yes/no)	96/53	114/677	180.797	0.000*	0.618	0.681
Tea (yes/no)	109/40	309/482	59.006	0.000*	0.374	0.541
Hypertension (yes/no)	74/75	365/426	0.624	0.429	<0.001	1.000
Diabetes (yes/no)	33/116	114/677	5.687	0.017*	<0.001	1.000
Hyperlipidemia (yes/no)	21/128	138/653	1.003	0.317	-	-
Brain trauma (yes/no)	4/145	31/760	0.533	0.465	<0.001	1.000
MoCA						
Abstract (0, 1, and 2 score)	25.5%/34.2%/40.3%	15.7%/37.2%/47.2%	7.931	0.019*	0.918	0.632
MoCA total	25.28 ± 2.659	25.34 ± 2.854	1.227	0.268	0.602	0.444
NTB						
Mapping	11.42 ± 3.513	11.49 ± 3.627	5.177	0.023*	0.593	0.447


*Data shown as mean ± SD unless otherwise stated, ^∗^*p* < 0.05.*

### Biochemical Indexes Between Alcohol and Non-alcohol Drinking Groups in the Sub-database

There were 572 individuals with blood biochemical tests from the whole database. The demographics, physical disease, lifestyle, and lipid profiles for alcohol (*n* = 100) and non-alcohol (*n* = 472) groups are listed in **Table 2**. The effects of these with differences between two groups were regressed. Through regular statistical analysis, a higher HDL plasma level was found in the alcohol consumption group than the non-alcohol consumption group (*p* < 0.05). Based on the normal range of HDL plasma levels, the subjects were divided into normal, high (male > 1.66 mmol/l, female > 1.75 mmol/l) and low (male < 1.04 mmol/l, female < 1.10 mmol/l) HDL grades, however, more subjects with abnormal total bilirubin (>17.1 μmol/L) were in the alcohol group than the non-alcohol group (*p* < 0.05). For balancing the sample size and gender bias in two groups, bootstrapping analysis was used and showed that there were no any positive findings (*p* > 0.05).

**Table 2:** Demography, life style, physical diseases, and lipid metabolism in non-dementia aging Han Chinese population with blood test.

	Alcohol (*n* = 100)	Non-alcohol (*n* = 472)	*F* or χ^2^	*p*-Value	Bootstrapping	
					Mean *F* or χ^2^	Mean *p*-value
Age (years)	69.84 ± 7.042	69.65 ± 7.527	0.052	0.819	0.821	0.536
Male/female	88/12	186/286	78.077	0.000*	<0.001	1.000
Education (years)	9.52 ± 3.642	9.81 ± 3.800	0.478	0.490	1.493	0.420
Smoking (yes/no)	66/34	80/392	104.440	0.000*	<0.001	1.000
Tea (yes/no)	75/25	174/298	48.820	0.000*	2.574	0.269
Hypertension (yes/no)	52/48	208/264	2.094	0.148	0.613	0.630
Diabetes (yes/no)	18/82	71/401	0.549	0.459	0.442	0.706
Hyperlipidemia (yes/no)	10/90	63/409	0.831	0.362	0.761	0.628
Brain trauma (yes/no)	1/99	16/456	1.634	0.201	0.249	0.801
High density lipoprotein (HDL)						
HDL (mmol/L)	1.185 ± 0.332	1.169 ± 0.291	10.719	0.001*	2.175	0.287
HDL (normal/high/low)	55%/37%/8%	51.9%/42.6%/5.5%	0.008	0.928	3.061	0.326
**Total bilirubin**						
Total bilirubin (μmol/L)	11.612 ± 4.786	10.232 ± 4.862	3.260	0.072	1.117	0.478
Total bilirubin (normal/high)	85%/15%	93.4%/6.6%	6.834	0.009*	0.374	0.747


*HDL range (male: 1.04–1.66 mmol/L, female: 1.1–1.74 mmol/L), total bilirubin range (3.4–17.1 μmol/L).Data shown as mean ± SD unless otherwise stated, ^∗^*p* < 0.05.*

### Brain Structural Differences Between Alcohol and Non-alcohol Consumption Groups in the Sub-database

**FIGURE 1:**
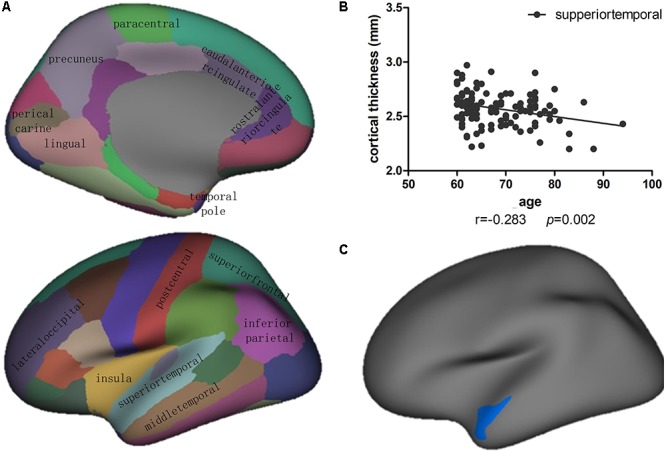
**(A)** Correlation and linear regression analysis showed that the cortical thickness of multiple cortical regions were significantly correlated with age in 114 cognitively normal aging subjects with non-alcohol consumption (*p* < 0.05). **(B)** The correlation of superiotemporal thickness with age showed as the scattered figure. **(C)** Vertex-based analysis of cortical thickness revealed that the left superiortemporal cluster showed significant difference between alcohol (*n* = 25) and non-alcohol (*n* = 49) consumption group without female subjects. Blue indicates that the cortical thickness of a cluster in the alcohol group was thinner than in the non-alcohol group (*p* < 0.05).

There were 141 individuals including males and females with brain MR images from the whole databasle database. Correlation analysis found that there were cortical thickness of multiple cortical regions were negatively associated with age, including lobes of superiotemporal, inferioparietal, precuneus, and etc. (*p* < 0.05) (**Figure 1A**). Among multiple age sensitive regions, the correlation of superiotemporal thickness with age was shown in **Figure 1B**.

The demographics, physical diseases, lifestyle, and subcortical structure volumes for the alcohol (*n* = 25) and non-alcohol (*n* = 49) groups without female subjects are listed in **Table 3**. There were differences between groups in education and smoking, so the effects of those were regressed. The subcortical structures volume were considered as the proportion of total intracranial volume. There were no differences in the volume of bilateral hippocampus or amygdala between groups (*p* > 0.05). Meanwhile, analysis of brain cortical anatomy showed the cortical thickness of the left superiortemporal cluster in alcohol group was significantly lower than non-alcohol group (*p* < 0.05) (**Figure 1C**).

**Table 3:** Demography, life style, physical diseases, and hippocampus volume in non-dementia aging Han Chinese population with brain MRI examination.

	Alcohol (*n* = 25)	Non-alcohol (*n* = 49)	*F* or χ^2^	*p*-Value
				
Age (years)	66.440 ± 5.083	69.490 ± 8.329	2.806	0.098
Male	25	49	-	-
Education (years)	9.520 ± 3.318	11.306 ± 2.867	5.773	0.019^∗^
Smoking (yes/no)	21/4	21/28	11.417	0.001^∗^
Tea (yes/no)	20/5	28/21	3.795	0.051
Hypertension (yes/no)	12/13	21/28	0.177	0.674
Diabetes (yes/no)	5/20	8/41	0.154	0.694
Hyperlipidemia (yes/no)	4/21	3/46	1.886	0.170
Brain trauma (yes/no)	1/24	3/46	0.146	0.703
Left hippocampus proportion (%)	0.240 ± 0.025	0.245 ± 0.027	0.505	0.479
Right hippocampus proportion (%)	0.256 ± 0.026	0.257 ± 0.029	0.006	0.940
Left amygdala proportion (%)	0.102 ± 0.014	0.102 ± 0.013	0.006	0.940
Right amygdala proportion (%)	0.112 ± 0.014	0.115 ± 0.015	0.457	0.501


*Data shown as mean ± SD unless otherwise stated, ^∗^*p* < 0.05.*

## Discussion

To our knowledge, this is the first study to explore the relationship of light-to-moderate alcohol drinking with cognitive function, biochemical indexes and brain anatomy in non-dementia aging Han Chinese population. First, we found no significant difference in cognitive function between alcohol and non-alcohol consumption group in the overall database. Second, we also found no significant difference in biochemical indexes between two groups in the sub-database. Third, there were multiple brain gyri were age sensitive, and only the cortical thickness of left superiotemporal cluster in alcohol group was significantly lower than non-alcohol group.

In the present study, we found that light-to-moderate alcohol consumption had no significant association with better or worse cognitive functions. There were many researchers thought that there were cognitive function benefits with light-to-moderate alcohol consumption. Lang et al. (2007) recruited 6005 individuals aged 50+ in England to analyze the relationship of alcohol consumption with cognitive function including word recall (10-word recall list), numerical reasoning, and time orientation, and found better cognition was associated with moderate alcohol drinking than with non-alcohol drinking. Reid et al. (2006) studied 760 adults aged 65 years or older to explore the effect of light-to-moderate drinking on cognitive function. They found that the cognitive outcome measures including Trails B, Symbol Digit and Hopkins Verbal Learning tests were better than a reference group of never and former drinkers (Reid et al., 2006). Our study was different with the above studies. The one of possible reason was the different cognitive assessment methods adopted. In the present research, MoCA test and Chinese version of the NTB were used to investigate elderly cognitive function, which was not involved in the above studies. Furthermore, there was a more important reason to explain the different conclusions, which was the diversity in drinking culture. Corley et al. (2011) thought that effects differed according to beverage type; wine consumption was associated with better verbal ability, but beer correlated with a poorer verbal ability. In Scotland, women’s alcohol intake was mostly derived from wine, and the proportion of wine and sherry consumption in men was high; however, in our study, there were more people who drank liquor (36.2%), yellow rice (37.6%), and beer (15.4%), with only 10.7% who drank wine in the Han Chinese population.

We analyzed the correlation of age with cortical thickness and found some age sensitive cortex such as superiotemporal, insula, superiorfrontal, and inferiorparietal lobes et al. Among multiple age sensitive regions, only the cortical thickness of the left superiortemporal cluster in alcohol group was significantly different from the non-alcohol group, which suggested that alcohol consumption was significantly associated with accelerated aging in left superiortemporal lobe. As is known, the superiortemporal gyrus forms part of Brodmann area 38, which is one of three gyri in the temporal lobe. The superiortemporal lobe is responsible for processing sounds and comprehension of language, and it also has been implicated as a critical structure in social cognition. We didn’t find the difference of language ability between two groups in the present study, it was possibly due to that we didn’t adopt professional language tests such as Boston diagnostic aphasia examination. Light-to-moderate alcohol consumption was associated with adverse brain outcome in our study, and it was similar with the previous studies. Lange et al. (2017) adopted brain MR imaging and the Alcohol Use Disorder Identification Test’s consumption part (AUDIT-C) in a study of 609 alcohol consuming adults, and found that higher AUDIT-C scores were linearly associated with thinner cortex in some brain regions. An observational cohort study of 550 participants with weekly alcohol intake and cognitive performance measured repeatedly over 30 years, showed that those drinking moderately had three times the odds of right hippocampal atrophy (Topiwala et al., 2017), which was different with our findings. We also analyzed the difference of subcortical structure volume proportion between two groups, especially the cognitive related structure including hippocampus and amygdala, and we didn’t get any positive findings. The probable cause could be that it was a longitudinal observation in Topiwala’s study but a cross-sectional observation in our study, and a further follow-up study would be continued.

Meanwhile, we didn’t find a significant difference in biochemical indexes between alcohol and non-alcohol groups. As we known, alcohol-induced liver injury and dyslipidemia is common in clinical (O’malley et al., 2015; Cho et al., 2017). In our research, the reason that levels of liver function and lipid metabolism indexes did not differ between the two groups was due to studying light-to-moderate alcohol intake, rather than binge drinking.

There were several factors that limited the findings of the present study. Because it had a cross-sectional study design, it could not show direct causality of alcohol consumption, whether beneficial or harmful. The factor limiting generalization of our study is that our sample size of alcohol consumers was much smaller than non-alcohol consumers, especially in the cortical anatomy analysis, due to a low prevalence of alcohol drinking in the cognitively normal elderly in the Shanghai community. There was gender bias even though gender as a factor was regressed in the tests of cognitive function and blood biochemistry. In the analysis of cortical anatomy, we eliminated women to avoid the gender bias because of only two female subjects involved in the alcohol group. It is also because women are eliminated, the analysis of MRI is not comprehensive enough to cover both genders population based on the limited sample size in our study.

## Conclusion

Our results showed that light-to-moderate alcohol consumption was not associated with better or worse cognitive functions and abnormal biochemical indexes, however, associated with thinner cortex of the left superiortemporal gyrus in a non-dementia aging Han Chinese population.

## Ethics Statement

This study was carried out in accordance with the recommendations of ‘Shanghai Mental Health Center ethical standards committee on human experimentation’ with written informed consent from all subjects. All subjects gave written informed consent in accordance with the Declaration of Helsinki. The protocol was approved by the ‘Shanghai Mental Health Center ethical standards committee’. All subjects also gave written informed consent for the publication of this case report.

## Author Contributions

LS and HX analyzed the data and wrote the manuscript. WL, JN, QQ, YL, and YF evaluated the subjects and collected the data. XL and SX designed the experiment and monitored the quality of the experiment. ZY analyzed the MR imaging data and drafted imaging part of the manuscript. JZ analyzed the statistics data and drafted the statistics part of the manuscript.

## Conflict of Interest Statement

The authors declare that the research was conducted in the absence of any commercial or financial relationships that could be construed as a potential conflict of interest.

## Footnotes

1 http://surfer.nmr.mgh.harvard.edu/
